# Bridging single‐species research and mixture reality: Emerging contaminants fate and transport in vadose zones

**DOI:** 10.1002/jeq2.70177

**Published:** 2026-04-06

**Authors:** Aaron Lee M. Daigh

**Affiliations:** ^1^ Department of Agronomy & Horticulture University of Nebraska‐Lincoln Lincoln Nebraska USA; ^2^ Department of Biological Systems Engineering University of Nebraska‐Lincoln Lincoln Nebraska USA; ^3^ Department of Environmental, Agricultural, and Occupational Health University of Nebraska Medical Center Omaha Nebraska USA

## Abstract

Vadose zones serve as interfaces controlling contaminant transport from the land surface to underlying aquifers. While traditional research has largely focused on individual contaminant species, real‐world contamination often involves complex mixtures containing dozens to thousands of chemical species. This disconnect creates considerable challenges for predicting contaminant behavior, assessing environmental risks, and developing effective remediation strategies. This review examines complex contaminant mixtures in vadose zones, with emphasis on per‐ and polyfluoroalkyl substances, pharmaceuticals and personal care products, and hydraulic fracturing fluid additives and contaminants. We synthesize recent advances in understanding mixture behavior, including competitive sorption at air‐water interfaces and solid surfaces, co‐facilitated transport mechanisms, and transformation dynamics under co‐contaminant conditions. Field observations from contaminated sites reveal that mixture effects alter transport rates, retention capacities, and degradation pathways relative to single‐species predictions. While vadose zones function as persistent secondary sources, transient saturation conditions can enhance contaminant transport by an order of magnitude relative to constant‐flow predictions. Current modeling frameworks remain limited in their capability to account for complex physicochemical interactions in contaminant mixtures, particularly under transient flow and heterogeneous environments. We identify and describe six priority areas for new or enhanced research to bridge current knowledge gaps: mixture sorption and competitive transport, transformation products and reaction pathways, field‐scale validation studies, multi‐mechanism remediation technologies, integrated mixture toxicity assessment, and climate change and evolving land use impacts.

## Core Ideas

- Mixture behavior in vadose zones may diverge from single‐species predictions.
- Competitive sorption at air‐water interfaces can alter contaminant mobility in mixtures.
- Transient flow enhances contaminant transport as compared to constant‐saturation predictions.
- Vadose zones serve as persistent secondary sources sustaining groundwater contamination.
- Mixture‐focused research frameworks are needed to advance vadose zone science.

## Plain Language Summary

Soil acts as a natural filter that protects groundwater, a key drinking water source for billions of people, from pollution. Scientists usually study how one chemical at a time moves through soil, but real pollution often contains hundreds or thousands of different chemicals mixed together. This review looks at how these chemical mixtures behave in soil, focusing on industrial chemicals, medicines, and fluids from oil and gas drilling. We found that chemicals in mixtures interact with each other, causing some to move faster and others slower than expected when studied alone. We also found that polluted soil can keep releasing chemicals into groundwater for decades. Current predictions based on single‐chemical studies may greatly miss the mark on actual risks. We identified six priority areas where new or improved research is needed to better understand and manage these complex pollution problems in the real world.

**Abbreviations**
HFFA‐HFFC: hydraulic fracturing fluid additives and contaminants
PFAS: per‐ and polyfluoroalkyl substances
PFOA: perfluorooctanoic acid
PFOS
: perfluorooctanesulfonic acid
PPCP: pharmaceuticals and personal care products

## 1 INTRODUCTION

The vadose zone, defined as the variably saturated depth extending from the land surface to a subsurface water table, is a critical component of the terrestrial environment that controls the fate and transport of contaminants released at or near the earth surface (Stewart et al., 2025). As the final barrier protecting groundwater resources that serve as drinking water sources for billions of people worldwide, understanding contaminant behavior in this zone is essential for effective environmental management. Historically, vadose zone research has concentrated on traditional pollutants, including agricultural fertilizers and pesticides, heavy metals, radionuclides, and petroleum hydrocarbons. For these contaminant classes, substantial progress has been achieved in characterizing transport mechanisms, developing predictive models, and implementing remediation strategies. However, in recent decades, a new generation of environmental contaminants has emerged as topics of scientific inquiry and regulatory concern (H. Chen, Gao et al., 2025; Stewart et al., 2025).

Per‐ and polyfluoroalkyl substances (PFAS), pharmaceuticals and personal care products (PPCP), and hydraulic fracturing fluid additives and contaminants (HFFA‐HFFC) represent three prominent classes of emerging contaminants that pose unique challenges for vadose zone science (Stewart et al., 2025). These contaminant classes share several common characteristics that distinguish them from traditional pollutants (Buck et al., 2011; Koelmel et al., 2021; Stewart et al., 2025; Wollin et al., 2020; Xiao, 2017; Xiao et al., 2017; Yin et al., 2017):

1. Each class encompasses thousands of individual chemical species, many of which remain unidentified or poorly characterized.
2. These contaminants exhibit diverse physicochemical properties spanning wide ranges of molecular weights, functional groups, degree of hydrophobicity, and charge states.
3. They are typically released into the environment as complex mixtures rather than as isolated compounds, and they frequently co‐occur with contaminants from other classes due to overlapping sources and pathways.

Despite these environmental contaminants occurring as complex mixtures, the overwhelming majority of research remains focused on individual contaminants. While scientifically tractable and valuable for elucidating fundamental processes, the predominant focus on single species (and even studies examining simple mixtures of only two or three species) still leaves a disconnect between research and the physical reality of many real‐world situations of contamination involving dozens to hundreds of co‐occurring chemicals (H. Chen, Gao et al., 2025; Stewart et al., 2025). Single‐species studies provide limited insight into competitive sorption, co‐facilitated transport, synergistic degradation, and mixture toxicity effects that are innate to many contamination scenarios. Practical approaches for systematically investigating higher complexity mixtures do exist, including pseudospecies grouping that reduces multicomponent systems to effective representative constituents (Gaganis et al., 2004), bioanalytical tools that quantify aggregate mixture effects without requiring identification of all individual components (Escher et al., 2020), and tiered assessment frameworks (e.g., priority “constituent blocks” or surrogates) for substances of unknown or variable composition (Salvito et al., 2020); these strategies are discussed in Sections 4. Recognition of these mixture effects has prompted increased scientific attention to contaminant mixtures across multiple disciplines in the past couple of decades (Reddy, 2011). However, realignment to elucidate the behavior of chemical mixtures in soils and the vadose zone remain largely absent. The gap between single‐species research and mixture‐based contamination sites has important implications for risk assessment, where predictions based on individual contaminants may substantially underestimate or overestimate actual environmental behavior and risk (H. Chen, Gao et al., 2025; Cipullo et al., 2018; Cremer et al., 2016; Kookana et al., 2022). It also affects remediation planning, as strategies optimized for single contaminants may prove ineffective or counterproductive when applied to complex mixtures (H. Chen, Gao et al., 2025).

This review represents a focused and in‐depth extension of topics briefly introduced in Section 7 on “Emerging Contaminants in the Vadose Zone” of Stewart et al. (2025). We contributed that section's content on contaminant mixtures (i.e., PFAS, PPCP, and HFFA‐HFFC); however, space constraints in that broad‐scope review article allowed for only their brief mention and precluded comprehensive treatment of such complex mixture interactions and transport that characterize many vadose zone contamination sites. Here, we address this limitation by providing detailed analysis, mechanistic discussion, and research prioritization on the importance of contaminant mixtures. The primary objective of this review is to synthesize current understanding of complex contaminant mixtures in vadose zone environments, with particular emphasis on PFAS, PPCP, and HFFA‐HFFC. Specifically, we examine sources and pathways through which contaminant mixtures enter vadose zones, explore the fate and transport processes governing mixture behavior, evaluate current modeling capabilities and limitations, present field observations (select contamination site studies) that illustrate mixture effects, and identify critical knowledge gaps that we think require future research to advance vadose zone science toward more realistic and applicable frameworks for complex environmental contamination situations (i.e., contaminant mixtures).

## 2 THE CONTAMINANT MIXTURE PROBLEM IN VADOSE ZONES

**FIGURE 1:**
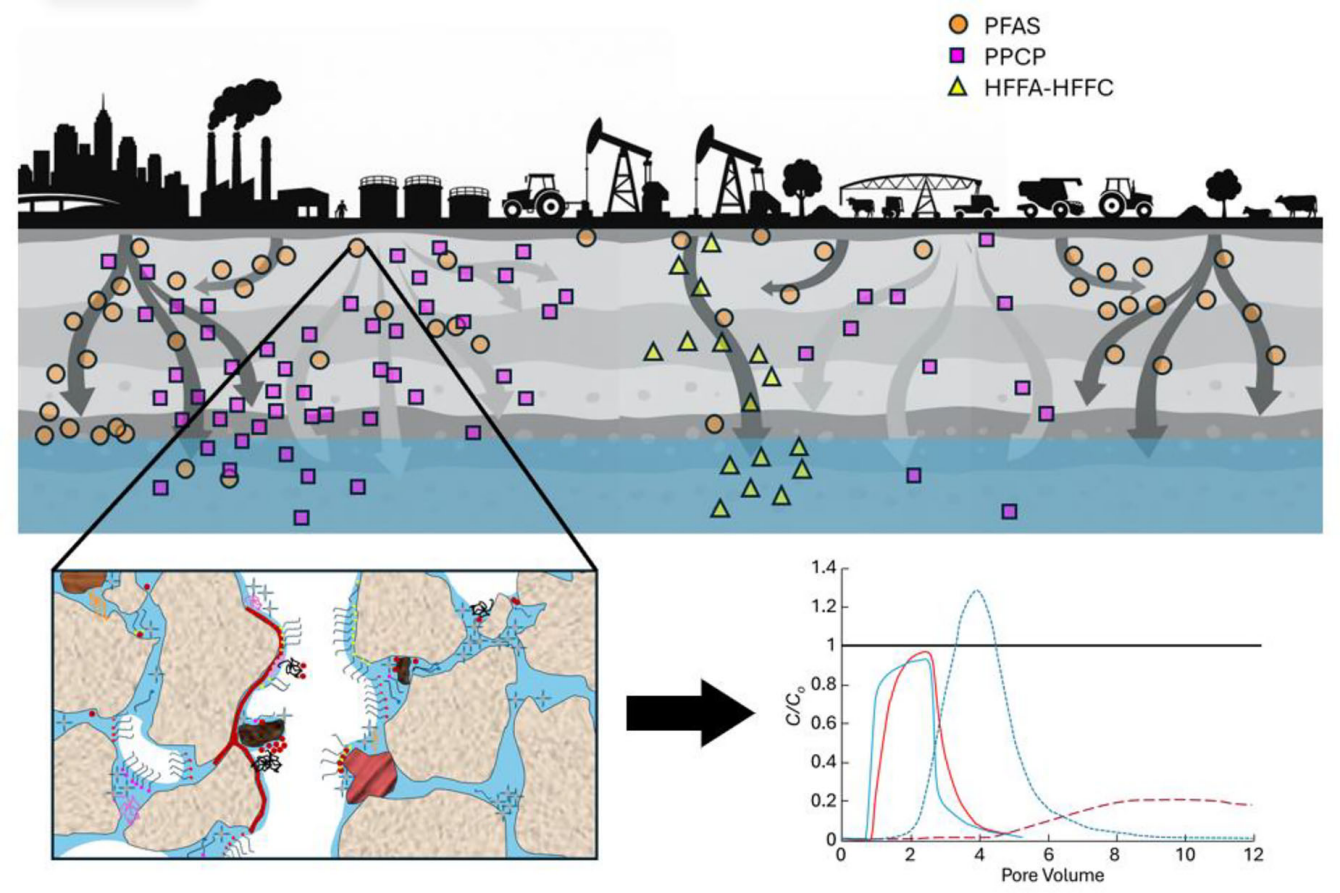
Conceptual diagrams of common emerging contaminant sources, pore‐scale complexity of contaminant mixtures, and breakthrough curves for contaminant transport through vadose zones. Urban and industrial wastewater treatment facilities; oil and gas extraction from hydraulic fracturing operations; and agricultural practices, including biosolids and manure applications to fields, irrigation with wastewaters, and livestock production, are common source pathways for per‐ and polyfluoroalkyl substances (PFAS), pharmaceuticals and personal care products (PPCP), and hydraulic fracturing fluid additives and contaminants (HFFA‐HFFC) to enter vadose zones (top diagram). Many sources of these emerging contaminants result in complex mixtures occupying soil and vadose zone pore spaces (bottom left diagram; inspired by and created based on Figure 1 from Zhang and Guo (2024) and the graphical abstract from W. Zhang et al. (2025); used with permission from publisher). These mixtures may contain dozens to thousands of chemical species, which may undergo competitive sorption or exchange sites on water‐mineral and air‐water interfaces (among species of the emerging contaminant mixture itself and with antecedent soil contaminants such as heavy metals, prior chemical spill residuals, etc.); co‐transport by complexation with other compounds and mobile colloids; and numerous transformation and degradation pathways and kinetics. Due to the complexity introduced by mixtures of emerging contaminants, the fate and transport of these chemicals and risk to water quality can vary substantially, with breakthrough concentrations and timelines differing substantially from single‐species predictions (bottom right diagram; data in breakthrough curves from Garza‐Rubalcava et al. [2025]; used with permission from publisher).

Contaminant mixtures enter vadose zone environments through numerous pathways reflecting the widespread use and disposal of synthetic chemicals in modern society (Figure 1). Although many of these pathways are routinely described in the literature, we briefly review some of the main pathways in this section for PFAS, PPCP, and HFFA‐HFFC as well as their mixture diversity. Then, we provide a more in‐depth evaluation on these mixture's fate and transport processes (Section 3), modeling and predicting their behaviors (Section 4), and highlighting observations from select contamination site studies (Section 5).

**Core Ideas**

- Mixture behavior in vadose zones may diverge from single‐species predictions.
- Competitive sorption at air‐water interfaces can alter contaminant mobility in mixtures.
- Transient flow enhances contaminant transport as compared to constant‐saturation predictions.
- Vadose zones serve as persistent secondary sources sustaining groundwater contamination.
- Mixture‐focused research frameworks are needed to advance vadose zone science.

### 2.1 Sources and pathways of contaminant mixtures

Urban areas represent major sources of PFAS and PPCP due to their ubiquitous presence in consumer products, industrial applications, and healthcare systems (Stewart et al., 2025). Municipal wastewater treatment plants, while effective at removing many traditional pollutants, typically achieve only partial removal of PFAS and PPCP compounds. Consequently, treated effluents discharged to surface waters or applied for irrigation may contain residual concentrations of these contaminants as complex mixtures reflecting their diverse sources (Jha et al., 2021). Wastewater biosolids, the solid byproduct of treatment processes, concentrate many organic contaminants through sorption to particulate matter. When biosolids are applied to agricultural lands as soil amendments to provide nutrients and organic matter, they simultaneously introduce mixtures of PFAS, PPCP, microplastics, antibiotic resistance genes, and trace metals to vadose zone environments (Jha et al., 2021).

Agricultural operations also generate contaminant mixtures through multiple pathways. Livestock production facilities release pharmaceuticals, hormones, growth regulators, and antibiotics through animal excrement, creating complex mixtures that enter soils when manure is applied as fertilizer (Jha et al., 2021). The composition of these mixtures varies with animal type, production practices, and veterinary pharmaceutical usage patterns. Similarly, companion animal waste in urban and suburban environments contributes PPCP mixtures to vadose zones through various disposal and runoff pathways. Irrigation with reclaimed wastewater or surface water contaminated by upstream discharges provides another pathway for introducing PPCP and PFAS mixtures to agricultural soils (Jha et al., 2021; Thalmann et al., 2025; Zentner et al., 2015). The chronic, repeated application of contaminated irrigation water or biosolids over years to decades can lead to substantial accumulation of persistent contaminants in vadose zones, even when individual application concentrations are low (Maddela et al., 2022; Zentner et al., 2015).

Industrial and military activities represent concentrated sources of PFAS mixtures, particularly at sites where aqueous film‐forming foams (AFFF) have been used for firefighting training and emergency response. AFFF formulations contain complex mixtures of PFAS compounds spanning multiple chemical classes with diverse chain lengths and functional groups (McGarr et al., 2023; Nickerson et al., 2021). When released to the environment, these AFFF‐derived mixtures infiltrate through vadose zones carrying both target PFAS compounds and numerous precursor species that subsequently transform to generate additional PFAS over time (Harris et al., 2025; ITRC, 2023). The result is dynamic mixture compositions that evolve through chemical and biological transformation processes occurring within the vadose zone itself (McGarr et al., 2023; Shea et al., 2025).

The oil and gas extraction industry introduces HFFA‐HFFC mixtures to vadose zones through accidental spills, well blowouts, leaks from storage and transport infrastructure, and intentional discharge (A. Klaustermeier et al., 2016). Hydraulic fracturing operations inject massive volumes of fluids containing diverse chemical additives to enhance fracture propagation and hydrocarbon recovery (McLaughlin et al., 2016). Flowback and coproduced waters returning to the surface carry not only the injected HFFA but also numerous contaminants mobilized from deep geological formations, including dissolved salts, heavy metals, and naturally occurring radioactive materials (Shrestha et al., 2017). Documented spills of these fluids introduce complex mixtures under extreme geochemical conditions, including near‐saturating salt concentrations that profoundly affect contaminant behavior in vadose zones. A comprehensive review of remediation challenges in the Williston Basin documented how brine impacts soil physical, chemical, and biological properties, often requiring extensive intervention to restore soil function (Green et al., 2020).

Landfill leachate represents another significant source of complex contaminant mixtures to vadose zones. Modern municipal solid waste contains synthetic organic chemicals from countless consumer products, pharmaceuticals, pesticides, and industrial materials. As water percolates through landfills, it dissolves and mobilizes diverse contaminants, creating leachate mixtures that may contain hundreds of organic compounds spanning multiple chemical classes alongside heavy metals, ammonia, and dissolved organic matter (DOM) (Kjeldsen et al., 2002; N. Zhang et al., 2024). Leachate composition evolves over time as landfills age and water decomposition progress, with younger landfills generating leachates enriched in volatile fatty acids and biodegradable organics while older landfills produce more recalcitrant humic substances carrying persistent organic contaminants (Kjeldsen et al., 2002). Emerging comtaminants, including PPCP and endocrine‐disrupting compounds, have been identified as significant constituents of municipal landfill leachates (Eggen et al., 2010), and PFAS are now recognized as widespread leachate contaminants due to disposal of PFAS‐containing products (Lang et al., 2017). When leachate migrates through underlying soils and vadose zones, these complex mixtures interact with soil components through sorption, degradation, and co‐transport processes that are difficult to predict from single‐species investigations.

### 2.2 Chemical diversity and mixture complexity

The chemical diversity encompassed within individual contaminant classes creates substantial complexity for understanding vadose zone behavior (Figure 1). The PFAS family alone includes thousands of individual compounds characterized by carbon‐fluorine chains of varying lengths combined with diverse functional head groups (Buck et al., 2011; Wang et al., 2017). Long‐chain PFAS containing seven or more perfluorinated carbons, such as perfluorooctanoic acid (PFOA) and perfluorooctanesulfonic acid (PFOS), dominated production and use for decades. Following regulatory restrictions on long‐chain compounds, beginning with voluntary industry phase‐outs (2000; 3M Company), the USEPA PFOA Stewardship Program (2006–2015), and listing of PFOS (2009) and PFOA (2019) under the Stockholm Convention (ITRC, 2023), industry has shifted toward short‐chain PFAS containing fewer than seven perfluorinated carbons, as well as compounds with cationic, zwitterionic, and nonionic functional groups in addition to the traditional anionic carboxylate and sulfonate moieties (Nguyen et al., 2020; Post, 2021; USEPA, 2016; Xiao et al., 2019). As a result, contemporary contamination sites may contain legacy long‐chain species, replacement short‐chain compounds, and transformation products generated from precursor molecules, creating dynamic mixture compositions spanning wide ranges of physicochemical properties. Recent analytical advances have identified >3000 PFAS compounds in commercial and environmental samples, far exceeding the dozens of target analytes routinely monitored (Lyu et al., 2022). This chemical diversity spans anionic, cationic, zwitterionic, and nonionic species with perfluoroalkyl chain lengths ranging from C2 to C16 or longer, creating mixture complexity that challenges both analytical characterization and transport prediction (Lyu et al., 2022).

Pharmaceuticals and personal care products encompass an even broader chemical space, including antibiotics, analgesics, hormones, antidepressants, stimulants, anticonvulsants, lipid regulators, and compounds used in personal hygiene products. The chemical structures and properties of PPCP span from highly polar, ionizable compounds to relatively hydrophobic neutrals. Many PPCPs undergo partial metabolism in humans and animals prior to excretion, and additional transformation occurs during wastewater treatment, generating degradation products that co‐occur with parent compounds in environmental mixtures (Yin et al., 2017). Transformation products frequently occur at concentrations exceeding their parent compounds and may exhibit greater persistence or toxicity, yet analytical methods typically target parent compounds while overlooking these derivatives (Escher et al., 2020). This analytical blind spot creates uncertainty regarding actual mixture composition and combined effects in vadose zones. Transformation products warrant particular research attention, as they may exhibit comparable or even greater toxicity and environmental persistence relative to parent compounds yet remain largely uncharacterized in environmental studies (Escher & Fenner, 2011; Fenner et al., 2013). Antibiotic resistance genes, while not chemical contaminants per se, represent an emerging concern closely associated with PPCP mixtures, particularly in environments receiving livestock manure or biosolids (Cycon et al., 2019; He et al., 2020; Stewart et al., 2025).

Hydraulic fracturing fluid additives include surfactants, friction reducers, biocides, corrosion inhibitors, scale inhibitors, and numerous other compounds used to optimize fracturing operations. Over 1100 distinct HFFA have been documented in industry databases, though many remain undisclosed as proprietary formulations (Wollin et al., 2020). The HFFA used vary substantially among different geological formations and operating companies, creating site‐specific mixture compositions. Flowback and coproduced waters add geogenic contaminants to the mixture, including variable concentrations of salts approaching solubility limits, heavy metals, radionuclides, and DOM from deep formations (Shrestha et al., 2017). The combination of anthropogenic HFFA and geogenic HFFC creates mixtures under extreme geochemical conditions rarely encountered in typical environmental systems.

Beyond these three major contaminant classes, vadose zone scientists increasingly recognize additional emerging contaminants that frequently co‐occur in complex mixtures (Figure 1). Microplastics and nanoplastics (particles <5 mm and <1 µm, respectively) are now nearly ubiquitous in environmental systems (Maddela et al., 2022). These particles can sorb hydrophobic organic contaminants, including PFAS and PPCP, serving as vectors for co‐transport through vadose zones (Llorca et al., 2018; Zhou et al., 2022). Engineered nanoparticles from consumer products and industrial applications represent another emerging contaminant class capable of co‐transport with sorbed organic chemicals (Chrysikopoulos & Fountouli, 2023). Tire wear particles, containing diverse organic additives and metals, contribute additional complexity to urban contaminant mixtures (Federico et al., 2023). The recognition that these various emerging contaminants frequently co‐occur emphasizes the need for mixture‐focused research approaches. Global soil estimates indicate that PFAS concentrations reach µg/kg levels across multiple continents, while microplastics accumulate at one to >10,000 particles/kg in agricultural soils receiving biosolids or plastic mulch (Maddela et al., 2022). Along with these contaminants, other additives, including phthalates and biphenyls, along with novel flame retardants, may co‐occur, creating multi‐class mixtures with diverse physicochemical properties and interactive mechanisms.

Overall, these examples clearly demonstrate the vast chemical diversity and mixture complexities that may be present in soils and vadose zones exposed to contamination. This daunting situation likely explains why vadose zone science has predominantly continued to focus on single species (or simple two‐ and three‐species mixtures) in the scientific literature. However, the chemical space created by these diverse compounds and their potential interactions is too vast for the traditional and exhaustive single‐species characterization with current analytical and modeling approaches. This necessitates the discipline to make strategic decisions about which subsets of contaminants to target and which processes to prioritize in research and monitoring programs to allow more direct and accurate representation of the complex mixtures within which they co‐occur.

## 3 FATE AND TRANSPORT PROCESSES FOR CONTAMINANT MIXTURES

Despite characterization challenges, some progress in recent years has been made in understanding the fundamental fate and transport processes governing contaminant mixture behavior in vadose zones, which we describe below. Specifically, the following sections examine competitive sorption, co‐facilitated transport, and transformation dynamics for mixture situations.

### 3.1 Competitive sorption and interfacial processes

**FIGURE 2:**
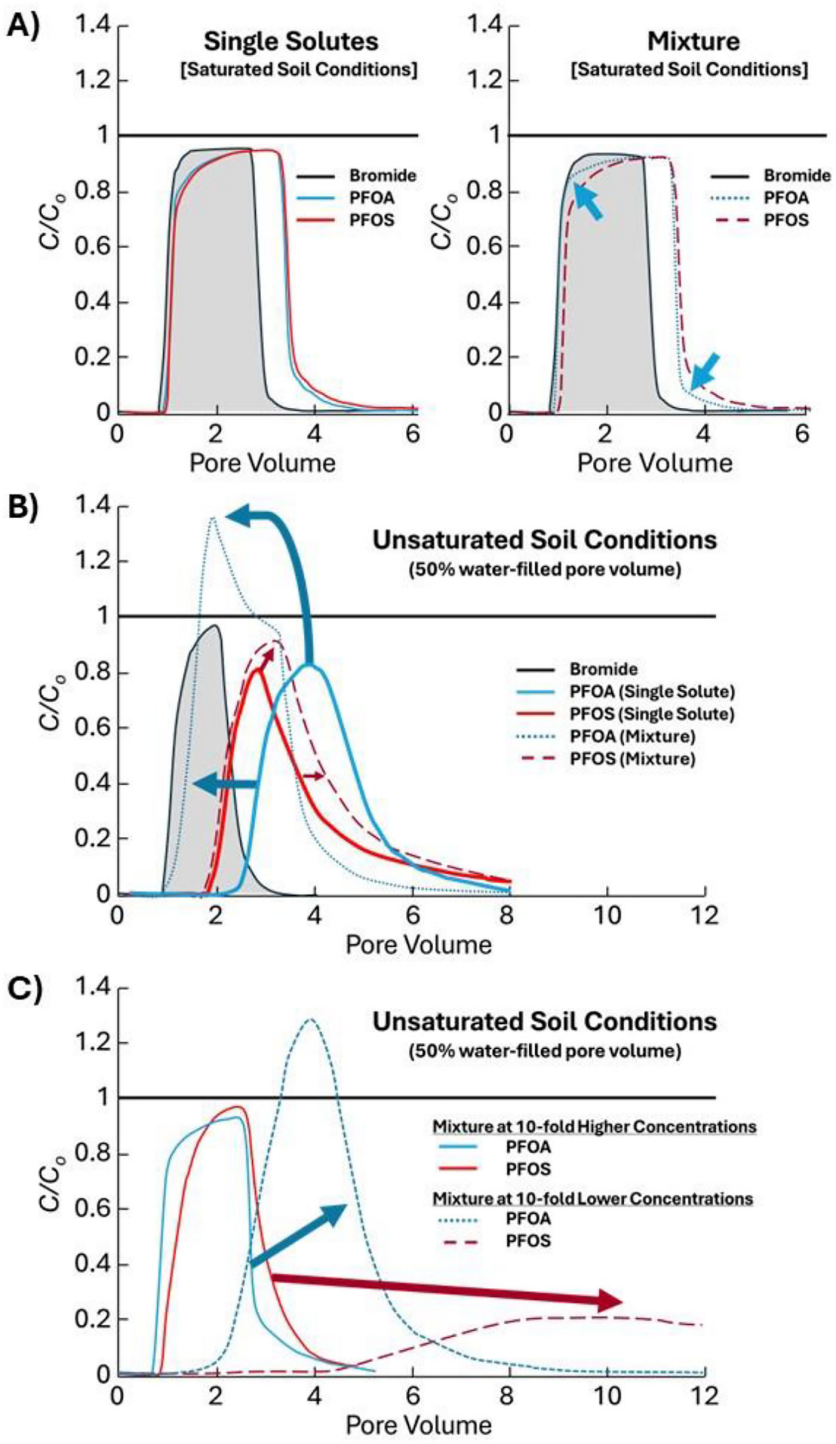
Chemical breakthrough curves of perfluorooctanoic acid (PFOA) and perfluorooctanesulfonic acid (PFOS) as single species and mixtures under various conditions (i.e., source concentrations and water‐filled pore space percentage). Graphs are the fitted curves for measured data. Under saturated conditions, PFOA and PFOS display similar breakthrough patterns to each other when occurring in isolation (single solute) (A, left panel), whereas PFOS displays some competitive sorption against PFOA, when occurring as a mixture with PFOA arriving and ending slightly earlier as pointed out with the blue arrows (A, right panel). Measured bromide breakthrough curves are shown and used to represent non‐reactive, conservative transport in the soil (A). However, differential lag times emerge, and competitive sorption is evident in unsaturated conditions (50% water‐filled pore space) with chromatographic peaking in mixtures (i.e., C/C_o_ > 1), which can occur with advancing displacement processes such as PFOS displacing sorbed PFOA (on both mineral‐water and air‐water interfaces) repeatedly as the solution advances through the soil (B). These competitive sorption processes of the mixture induce earlier breakthrough at higher concentrations than the source mixture for PFOA as compared to when present as a single solute (B). Similar to trends observed for mixtures in saturated soil, 10‐fold higher concentrations of the PFOA‐PFOS mixture induced nearly similar breakthrough curves with some slight patterns of competitive sorption (C, solid lines). However, notable differences in concentrations (including chromatographic peaking) and lag times occurred among PFOA and PFOS (panel C, dashed lines) when the source mixture was 10‐fold lower in concentrations as compared to panel B. All graphics in panels A, B, and C were recreated from data in Figures 4, 5, and 6, respectively, of Garza‐Rubalcava et al. (2025); used with permission from publisher.

Sorption processes exert primary control over contaminant transport rates and attenuation capacities in vadose zones. For single‐species systems, sorption is commonly described using equilibrium distribution coefficients or isotherm models parameterized from batch experiments and chemical breakthrough curves (Figure 2). When multiple contaminants are present simultaneously, however, competition for finite sorption sites can alter individual species behavior relative to single‐species predictions (Figure 2b,c). Competitive sorption becomes particularly important when contaminant concentrations approach or exceed the sorption capacity of available solid phases, air‐water interfaces, or organic matter domains (Figure 2b).

Recent experimental studies have documented competitive sorption effects in PFAS mixtures. As shown in Figure 2, Garza‐Rubalcava et al. (2025) observed that when PFOA and PFOS are present together in unsaturated porous media, they compete for adsorption at both solid‐phase surfaces and air‐water interfaces. PFOS, being more surface‐active due to its additional CF_2_ unit in the fluorinated tail and different head group chemistry (Fatima et al., 2025), tends to dominate interfacial adsorption, which can suppress PFOA uptake relative to single‐species behavior (Garza‐Rubalcava et al., 2025). Transport experiments conducted in unsaturated sand columns demonstrated that the presence of PFOS increased PFOA mobility compared to PFOA transport alone, consistent with competitive displacement from sorption sites (Garza‐Rubalcava et al., 2025). These competitive effects were successfully incorporated into a reactive transport model by explicitly accounting for interfacial area dynamics and competitive Langmuir‐type isotherms for air‐water interface adsorption (Garza‐Rubalcava et al., 2025). Similarly, binary mixture experiments with trapped gas bubbles showed enhanced PFOA breakthrough and reduced PFOA retardation in the presence of PFOS, with PFOA effluent concentrations temporarily exceeding influent concentrations prior to PFOS arrival due to competitive displacement from air‐water interfaces (Abraham et al., 2022). Similar competitive interfacial adsorption effects were observed in a study examining PFOS transport in multi‐component PFAS solutions, where the presence of co‐PFAS reduced PFOS retention at air‐water interfaces under high concentration conditions (i.e., 0.3 mg/L PFOA), though these competitive interactions were less apparent at lower concentrations (i.e., 0.1 mg/L PFOA) (Huang et al., 2022). Notably, the standard multiple‐component Langmuir model failed to predict this competitive adsorption behavior during transport, which indicates the need for more sophisticated modeling approaches for PFAS mixture systems (Huang et al., 2022). More broadly, the concentration dependency of competitive sorption effects extends beyond air‐water interfaces. Systematic batch and column experiments in fully saturated soil demonstrated that mixture effects on PFAS solid‐phase sorption and transport were significant only above approximately 20 µg/L and negligible at lower, environmentally relevant concentrations (Umeh et al., 2024).

The air‐water interface represents a particularly important sorption domain in vadose zones due to its large surface area and strong affinity for amphiphilic contaminants (Figure 1). Long‐chain PFAS compounds in particular accumulate preferentially at air‐water interfaces due to their hydrophobic perfluorocarbon chains and hydrophilic head groups, which drive them to interface regions where both moieties can achieve favorable energetic states (Brusseau, 2018; Lyu et al., 2018). This interfacial affinity generally increases with perfluoroalkyl chain length, as the air‐water interface partition coefficient (*K*_ai_) for long‐chain PFAS is notably greater than those for short‐chain species (Lyu et al., 2022). The total air‐water interfacial area in unsaturated porous media depends on water saturation, pore size distribution, and surface roughness, with estimates suggesting that interfacial area may retain 50% or more of total long‐chain PFAS mass in vadose zones (Brusseau, 2018; Silva et al., 2022). However, quantifying air‐water interfacial area remains challenging because principal measurement approaches (including aqueous interfacial tracer tests, synchrotron X‐ray microtomography, and thermodynamic methods) probe different interfacial domains and systematically yield different values for the same medium, with tracer‐measured areas approximately five times larger than tomographic estimates in natural media due to unresolved surface roughness (Brusseau et al., 2007). Directly measured datasets remain remarkably scarce, with nearly all obtained for glass beads or uniform sands rather than the heterogeneous, structured soils characteristic of field settings (Brusseau et al., 2024).

The magnitude of this interfacial retention is further modulated by pore water chemistry. Increasing ionic strength enhances air‐water interfacial adsorption by alleviating electrostatic repulsion among PFAS headgroups and increasing the activity of the hydrophobic tail in solution. Column experiments demonstrated that the retardation factor for PFOA approximately doubled across the tested ionic strength range under unsaturated conditions, whereas ionic strength had minimal effect under saturated conditions where solid‐phase adsorption was the sole retention mechanism (Lyu & Brusseau, 2020). Divalent cations such as Ca^2^^+^ exert a stronger effect than monovalent cations on interfacial adsorption, likely because their smaller hydrated radii allow greater penetration into the adsorbed monolayer (Lyu et al., 2022). Changes in pH, by comparison, have a more modest influence on PFAS retention at air‐water interfaces, with the air‐water interfacial adsorption contribution varying by only a factor of approximately 1.5 across a pH range of 5–8 (Lyu & Brusseau, 2020). However, pH can notably alter solid‐phase sorption of PFAS by changing the surface charge of soil organic carbon and clay minerals (Lyu et al., 2022). These solution chemistry effects are particularly relevant in mixture contexts because co‐contaminants and dissolved constituents in vadose zone pore water, including major ions from fertilizers, road salts, or co‐disposed wastes, may shift interfacial adsorption capacities in ways that single‐species laboratory studies conducted in simple electrolyte solutions do not capture.

Under transient flow conditions, changes in water saturation alter air‐water interfacial area, causing corresponding changes in contaminant partitioning that can either retard or accelerate transport depending on whether saturation is increasing or decreasing. Recent column experiments demonstrated that variably saturated flow conditions with periodic wetting and drying cycles can accelerate PFAS leaching by an order of magnitude relative to constant saturation conditions, despite significant interfacial retention (Shea et al., 2025). Therefore, transient flow effects, which are ubiquitous in natural vadose zones, need to be considered when predicting mixture transport. These transient effects are further complicated by hysteresis in the air‐water interfacial area, as interfacial area at a given degree of water saturation differs between drainage and imbibition (L. Chen & Kibbey, 2006). During imbibition events such as rainfall infiltration or groundwater table rise, collapsing air‐water interfaces release adsorbed PFAS into the aqueous phase, producing concentration pulses not captured by models parameterized from drainage‐only water retention data (Stults et al., 2024; Zeng & Guo, 2023). Despite this evidence, there appears to be no published PFAS transport model that directly incorporates soil water hysteresis’ relationship to the air‐water interfacial area.

Beyond affecting air‐water interface dynamics, transient boundary conditions fundamentally alter solute transport pathways in heterogeneous vadose zones. Using 2D numerical models of bimodal porous media, Cremer et al. (2016) demonstrated that infiltration‐evaporation cycles affect travel times and breakthrough behavior even when cycle‐averaged fluxes remain constant. Three distinct transport regimes emerged depending on infiltration intensity relative to material hydraulic conductivities: (1) at low intensities, upward and downward transport paths remain similar to stationary conditions; (2) at moderate intensities, divergent flow paths between coarse and fine materials lead to solute trapping and distinct breakthrough curve tailing; and (3) at high intensities, lateral advective‐diffusive transport across material interfaces accelerates downward movement. When material contrasts are weaker and pore structures are more tortuous, these lateral distribution effects become less pronounced, and the primary influence of dynamic boundary conditions is increased dispersion. These mechanisms likely contribute to observed field‐scale mixture transport complexity, particularly for contaminants exhibiting interfacial partitioning.

A comprehensive review of PFAS subsurface behavior confirms that air‐water interfaces represent the dominant retention mechanism for many PFAS in unsaturated porous media, with interfacial adsorption increasing with perfluoroalkyl chain length due to hydrophobic interactions, while zwitterionic and cationic PFAS exhibit strong solid‐phase retention driven by electrostatic mechanisms (Lyu et al., 2022). Field observations at AFFF‐impacted sites demonstrate that PFAS concentrations in vadose zone pore water can substantially exceed those in underlying groundwater, indicating that unsaturated zones function as persistent secondary sources sustained by slow desorption from air‐water interfaces.

Co‐contaminant interactions extend beyond competition among species within a single contaminant class. Heavy metals can alter PFAS sorption behavior through modification of soil surface charge and electrostatic interactions. Recent experiments showed that cadmium addition significantly increased PFOS sorption to soils due to cation bridging mechanisms, while arsenic presence reduced PFOS sorption through competitive effects (W. Zhang et al., 2025). Interestingly, these metal effects were more pronounced for long‐chain PFOS than for short‐chain PFOA or perfluorohexanesulfonic acid (PFHxS), suggesting that metal co‐contamination effects depend on both PFAS properties and metal speciation. Such observations underscore the complexity of predicting mixture behavior when contaminants from fundamentally different chemical classes interact through multiple mechanisms.

Certain PPCP compounds also exhibit affinity for air‐water interfaces, though typically weaker than PFAS. Column transport experiments with sulfonamide antibiotics in unsaturated sand demonstrated improved model predictions when air‐water interface adsorption was included alongside solid‐phase sorption in a dual‐domain conceptual model (Dai et al., 2020). The relative importance of interfacial versus solid‐phase sorption varies among PPCP depending on their hydrophobicity, surface activity, and ionization state, creating compound‐specific responses to changes in water saturation. Solution chemistry exerts analogous but mechanistically distinct controls on PPCP mobility. Because many PPCPs are ionizable, their speciation and sorption behavior are strongly pH‐dependent. For example, antipyretic drugs such as ibuprofen and indometacin showed enhanced mobility at higher pH due to increased electrostatic repulsion between dissociated anionic species and negatively charged soil surfaces (J. Chen et al., 2023). In the same study, Ca^2^^+^ reduced drug mobility relative to Na^+^ through cation‐bridging interactions that linked drug molecules to soil mineral surfaces (J. Chen et al., 2023). Natural vadose zone pore waters contain variable concentrations of major cations whose composition changes seasonally and with depth, suggesting that PPCP transport rates may fluctuate in response to pore water chemistry shifts even when other conditions remain constant.

When PPCP mixtures span ranges of these properties, differential partitioning among species during transient flow events can lead to chromatographic separation effects that alter mixture composition with depth and time. Controlled column experiments comparing pharmaceutical transport under unsaturated versus saturated conditions have demonstrated substantial differences in compound fate depending on water content (Dibyanshu & Scheytt, 2025). For instance, diclofenac exhibited high recovery (91%) under unsaturated conditions but lower recovery (45%) under saturated conditions due to enhanced degradation in the saturated medium, while carbamazepine maintained high mobility and persistence (>88% recovery) regardless of saturation state (Dibyanshu & Scheytt, 2025). Similar to predicting mixtures of contrasting chemical categories, such compound‐specific responses to saturation conditions induce additional complexity in predicting mixture behavior under the transient flow regimes that are innately characteristic in the active flux depths of vadose zones.

### 3.2 Co‐facilitated transport mechanisms

Colloids, broadly defined as particles between approximately 1 nm and 10 µm, serve as mobile sorbents that can facilitate contaminant transport when contaminants partition to colloid surfaces and are subsequently carried with flowing water. In vadose zone environments, colloids include clay minerals, metal oxides, organic matter, microorganisms, and anthropogenic particles such as microplastics and engineered nanoparticles. Colloid‐facilitated transport has long been recognized as an important process for hydrophobic organic contaminants and strongly sorbing metals (Bierbaum et al., 2023; Flury & Qiu, 2008; McCarthy & Zachara, 1989), but its role in mixture transport has received less attention.

Experimental studies have demonstrated PFAS association with various colloid types, including natural soil colloids and microplastics. When flow interruptions occur in saturated porous media, colloid remobilization can cause increases in pore water PFAS concentrations as colloid‐bound contaminants return to solution (Borthakur et al., 2021). The affinity of individual PFAS species for specific colloid types varies with chain length, functional group, and colloid surface properties, creating opportunities for selective co‐transport of certain mixture components. Microplastics in particular can sorb PFAS from solution, with smaller and more weathered particles exhibiting higher sorption capacities (Llorca et al., 2018). However, PFAS sorption to microplastics decreases in saline waters and at high pH, indicating that co‐transport potential depends on solution chemistry as well as contaminant‐colloid affinities.

Hydraulic fracturing fluids present unique examples of engineered colloid mobilization. Column experiments with flowback water demonstrated that HFFA enhanced mobilization of antecedent colloids in unsaturated sand, with 32%–36% of resident colloids being transported when flowback water was introduced (Sang et al., 2014). In contrast, deionized water mobilized <5% of colloids under comparable conditions. Increasing flow rates further enhanced colloid mobilization, suggesting that the surfactants and other additives in HFFA reduce colloid attachment to solid surfaces or air‐water interfaces. These mobilized colloids can co‐transport sorbed metals, radionuclides, and other strongly sorbing contaminants that would otherwise exhibit limited mobility. Thus, HFFA influences both their own transport and that of geogenic HFFC through colloid‐mediated mechanisms.

DOM, while not colloidal in the strict size definition, can similarly facilitate transport of hydrophobic contaminants through formation of mobile organic complexes. In landfill leachate and biosolids‐amended soils, elevated DOM concentrations can enhance mobility of hydrophobic PPCP and other organic contaminants that would otherwise sorb strongly to solid phases (Zhou et al., 2022). The composition and properties of DOM vary with source and decomposition stage, creating variable effects on different contaminant classes and species within those classes. Understanding these interactions remains challenging due to the chemical heterogeneity of both DOM and contaminant mixtures.

Engineered nanoparticles represent an emerging class of mobile colloids with potential to co‐transport sorbed contaminants. Experiments examining titanium dioxide nanoparticle transport in saturated and unsaturated sand columns showed that formaldehyde co‐transport was enhanced by nanoparticle presence, though nanoparticle attachment to air‐water interfaces and solid surfaces limited their overall mobility (Chrysikopoulos & Fountouli, 2023). The environmental relevance of nanoparticle‐facilitated transport depends on nanoparticle sources, their stability and mobility in vadose zones, and their affinities for co‐occurring contaminants. While current understanding remains limited, the increasing use of engineered nanoparticles in consumer products suggests this transport mechanism warrants further investigation.

### 3.3 Transformation and degradation in mixture systems

Biological and chemical transformation processes can attenuate contaminant concentrations in vadose zones, but their operation in mixture systems introduces complexity beyond single‐species scenarios. Microorganisms capable of degrading specific contaminants may exhibit enhanced, inhibited, or unaffected activity in the presence of co‐contaminants depending on mechanisms of interaction. Similarly, abiotic transformation processes including hydrolysis, oxidation, and photodegradation may be influenced by co‐occurring chemicals that alter reaction rates or pathways.

For PFAS mixtures, biodegradation potential remains limited due to the stability of carbon‐fluorine bonds under most environmental conditions. However, many PFAS exist as precursor molecules containing functional groups beyond the stable perfluoroalkyl chain (ITRC, 2023). These precursors can undergo biotransformation of their labile moieties, generating terminal PFAS such as PFOA and PFOS as transformation products (Harris et al., 2025; W. Zhang et al., 2021). Field observations at AFFF‐impacted sites indicate slow but persistent precursor transformation in vadose zones, sustaining groundwater contamination plumes decades after initial releases (Shea et al., 2025). The presence of co‐contaminants including high salinity from AFFF formulations or geogenic sources may inhibit microbial precursor degradation, extending persistence in vadose zones.

Pharmaceutical biodegradation exhibits compound‐specific patterns influenced by co‐contaminants and environmental conditions. Long‐term lysimeter studies irrigated with reclaimed wastewater containing PPCP mixtures showed that certain compounds, including carbamazepine, were largely retained or degraded in the upper vadose zone, while others, including some contrast agents, exhibited high mobility, with breakthrough occurring within years (Thalmann et al., 2025). As noted in Section 2.2, degradation products often retain biological activity and may exhibit toxicity comparable to or exceeding parent compounds, yet they are rarely monitored or included in risk assessments.

Mixture effects on biodegradation rates can be synergistic, antagonistic, or neutral depending on specific compound combinations and microbial community responses (Cedergreen, 2014). Co‐metabolism, where degradation of one compound is fortuitously enhanced by enzymes induced for degrading another compound, represents a potentially beneficial mixture effect (Suttinun et al., 2013). Conversely, some co‐contaminants may inhibit microbial activity through toxic effects, competitive enzyme inhibition, or alteration of favorable environmental conditions (Sandrin & Maier, 2003). Limited research has examined these mixture effects systematically, particularly under the complex geochemical and hydrological conditions characteristic of vadose zones (Cedergreen, 2014). Muñoz‐Vega et al. (2023) showed that soil biofilms, which form in redox‐active zones characteristic of managed aquifer recharge and irrigation systems, can influence pharmaceutical fate through enhanced sorption and biotransformation. Their column experiments with natural soils demonstrated that biofilms acted as additional sorption sites even for recalcitrant compounds such as carbamazepine, with the immobile phase fraction associated with biofilm structures contributing to compound retention beyond predictions based on soil properties alone. They also observed diclofenac degradation to be enhanced under denitrifying and manganese‐reducing conditions occurring within biofilm zones, implying a coupling between microbial community activity and redox conditions in controlling pharmaceutical fate.

Hydraulic fracturing fluid mixtures present extreme examples of co‐contaminant effects on degradation processes. Laboratory studies demonstrated that polyacrylamide friction reducers cross‐linked with glutaraldehyde exhibited reduced biodegradation compared to uncross‐linked polymers, and glutaraldehyde presence also inhibited degradation of polyethylene glycol surfactants (McLaughlin et al., 2016). The near‐saturating salt concentrations characteristic of flowback and coproduced waters severely limited biodegradation of all HFFA tested, effectively precluding natural attenuation as a viable remediation pathway without prior desalination. The degradation kinetics of individual HFFA constituents can also differ substantially depending on whether compounds are present in ultrapure water versus HF fluid mixtures. Triethanolamine, a common gel‐breaker aid, exhibited rapid degradation with approximately 90% loss within two weeks when applied to soils in ultrapure water but displayed biphasic decay patterns in soils treated with HF fluid mixtures, with degradation rates declining markedly after an initial rapid phase and half‐lives exceeding 30 days in some soils (Kookana et al., 2022). Such co‐contaminant effects on degradation kinetics have direct implications for transport predictions and natural attenuation assessments in HFFA‐HFFC contaminated systems. Such extreme mixture conditions fundamentally alter contaminant fate relative to that of individual species under moderate environmental conditions. Mixture interactions affecting contaminant fate extend beyond the extreme conditions characteristic of HFFA‐HFFC to more common environmental scenarios. Sorption of organic contaminants to microplastics alters their environmental mobility and bioavailability, while metal co‐contaminants can enhance or inhibit organic pollutant degradation depending on concentration and speciation (Maddela et al., 2022). These co‐contaminant effects remain poorly characterized for most emerging contaminant combinations under vadose zone conditions.

## 4 MODELING AND PREDICTING MIXTURE BEHAVIOR

### 4.1 Current modeling capabilities and limitations

Numerical models serve as essential tools for predicting contaminant fate and transport, supporting risk assessment, and guiding remediation design. Modern vadose zone transport models including HYDRUS, PFLOTRAN, and STOMP incorporate representations of water flow under variably saturated conditions, multi‐phase partitioning, advective‐dispersive transport, and various retention and transformation processes (Lichtner et al., 2015; Šimůnek et al., 2008; White & Oostrom, 2003). These models can accommodate multiple chemical species and simulate competitive sorption, sequential degradation reactions, and other processes relevant to mixture transport. However, their application to real contaminant mixtures faces substantial challenges related to parameter estimation, process representation, and computational demands.

Parameterizing transport models for contaminant mixtures requires sorption coefficients, degradation rate constants, and other parameters for each chemical species of interest. Estimation of transport parameters for PFAS in unsaturated media is complicated by pore‐scale mechanisms including immobile water zones and advective flow path tortuosity that influence observed breakthrough behavior and retardation, and analysis of experimental methods reveals that standard approaches may yield parameter estimates that do not fully capture these processes (Stults et al., 2022). These findings underscore the importance of carefully designed experimental protocols and appropriate model selection when characterizing PFAS transport for predictive applications. For emerging contaminants, these parameters often remain poorly characterized or entirely unknown, necessitating estimation from structure‐activity relationships, analog compounds, or limited experimental data. Parameter uncertainty propagates through model predictions, creating substantial ranges in predicted outcomes that limit model utility for decision‐making. When mixture complexity extends to dozens or hundreds of compounds, comprehensive parameterization becomes practically infeasible, forcing researchers to focus on a subset of priority compounds and implicitly neglect others.

Process representation presents another limitation. Most vadose zone models employ relatively simple descriptions of sorption, including linear or Freundlich isotherms for solid‐phase partitioning (Sima & Jaffé, 2021). While more sophisticated competitive sorption models exist, their parameters prove difficult to determine from batch experiments due to the large number of potential interactions in realistic mixtures. Air‐water interface sorption, increasingly recognized as critical for PFAS and certain PPCP, requires specialized model formulations and parameters rarely available (Silva et al., 2020). Sorption behavior also differs between short‐chain and long‐chain PFAS in ways that complicate simple modeling approaches (Fabregat‐Palau et al., 2021, 2025), with synthesis of literature data revealing that short‐chain anionic PFAS exhibit enhanced sorption relative to predictions based on organic carbon‐normalized correlations developed for long‐chain species (Brusseau, 2023). While the standard Koc‐foc approach may produce reasonable sorption estimates for long‐chain PFAS in soils with adequate organic carbon content, inorganic soil components, including silt and clay, contribute to short‐chain PFAS sorption, indicating that single‐mechanism sorption models inadequately represent the range of PFAS species present in environmental mixtures (Brusseau, 2023). Recent HYDRUS modifications have incorporated air‐water interfacial partitioning with competitive adsorption for PFAS mixtures, representing important progress (Silva et al., 2020; Vahedian et al., 2024). However, these advanced formulations require substantial parameterization effort and have seen limited application beyond research contexts.

Transformation processes in mixtures remain particularly challenging to represent mechanistically. First‐order degradation kinetics provide convenient mathematical descriptions that capture decay behavior when environmental conditions remain relatively constant and single contaminants degrade independently. In mixture systems where degradation rates depend on co‐contaminant effects, microbial community dynamics, and variable environmental conditions, first‐order approximations may poorly represent actual behavior. Few models incorporate mechanistic microbial processes or explicitly represent transformation product formation, limiting their ability to predict mixture evolution through time.

Field‐scale heterogeneity presents additional challenges for mixture transport modeling. Vadose zones exhibit spatial variability in hydraulic properties, degree of saturation, geochemical conditions, and microbial communities that create corresponding variability in contaminant sorption and transformation (Arshadi et al., 2024; Dechesne et al., 2014). Preferential flow paths through macropores, fractures, or coarse‐textured layers can substantially accelerate transport relative to predictions based on matrix flow in homogeneous media (Zeng & Guo, 2023). Transient boundary conditions add additional complexity to preferential flow predictions, as infiltration‐evaporation cycles can activate or deactivate different flow pathways depending on intensity and duration (Cremer et al., 2016; Wallis et al., 2022). Standard transport models employing constant flux boundary conditions may poorly represent mixture behavior under realistic temporal variability in surface fluxes. While conceptual models acknowledge these processes, incorporating them into predictive frameworks for mixture transport remains an active research area.

When mixture complexity extends to dozens or hundreds of poorly characterized compounds, as commonly occurs with hydraulic fracturing fluids, petroleum products, and biosolids‐derived contaminants of PPCP and PFAS, comprehensive component‐level characterization becomes infeasible. Risk assessment frameworks developed for substances of unknown or variable composition suggest hybrid approaches combining whole‐mixture testing with characterization of priority “constituent blocks” (e.g., surrogates) that share similar physicochemical properties (Salvito et al., 2020). Adapting these approaches to vadose zone transport prediction may enable mixture assessments without requiring exhaustive individual compound parameterization.

### 4.2 Recent advances and emerging approaches

Despite the challenges outlined above, recent years have seen meaningful advances in modeling contaminant mixtures under vadose zone conditions. Competitive sorption models originally developed for groundwater systems have been adapted and applied to unsaturated conditions (Garza‐Rubalcava et al., 2025; Selim & Zhang, 2013). Multi‐component Langmuir equations and related formulations allow explicit representation of competition for finite sorption sites among multiple species, though they require determination of maximum sorption capacities and competitive affinity parameters for each compound pair (Acharya et al., 2023; Garza‐Rubalcava et al., 2025). Column transport experiments with PFAS mixtures have successfully employed competitive isotherms combined with interfacial area models to predict transport under variable saturation conditions (Garza‐Rubalcava et al., 2025). While parameter demands remain substantial, these applications demonstrate feasibility for systems with limited numbers of priority contaminants. Recent modeling investigations have also noted the importance of representing realistic depth distributions of soil organic carbon and root zone processes for PFAS transport predictions, demonstrating that use of topsoil organic carbon measurements can overestimate retardation by more than threefold compared to profiles with organic carbon decreasing with depth (Biesek et al., 2024). Root water uptake was found to significantly slow PFAS movement through increased evapotranspiration while simultaneously causing evaporation‐induced concentration effects that elevated porewater PFAS concentrations, with these effects more pronounced in fine‐textured soils (Biesek et al., 2024).

Modeling frameworks that couple water flow, heat transport, and multi‐phase partitioning have been applied to volatile organic compound mixtures in vadose zones, accounting for temperature effects on vapor pressures and enabling simulation of mixture composition evolution through differential volatilization and transport (Gaganis et al., 2004). Though focused on petroleum hydrocarbons rather than emerging contaminants, these applications have implications for how process‐based models can capture mixture effects, including advective gas‐phase transport, temperature‐dependent partitioning, and first‐order biodegradation of individual mixture components. Model predictions showed reasonable agreement with field observations when carefully parameterized using site‐specific data, though simplified conceptual models often performed comparably to complex formulations for overall plume behavior (Gaganis et al., 2004).

Recent modeling studies of hydraulic fracturing spills have examined natural attenuation potential for HFFA‐HFFC mixtures in thick vadose zones. A HYDRUS‐based simulation considered 63 organic and inorganic chemicals representative of flowback water included dilution, dispersion, sorption, biodegradation, and radioactive decay in soil profiles overlying groundwater (Mallants et al., 2022). Model results suggested that deep vadose zones provide substantial natural attenuation for most individual chemicals, reducing concentrations below ecological risk thresholds before reaching groundwater. However, when direct toxicity assessments were employed to account for chemical mixture effects, the safe dilution factor required for 95% species protection proved 1.8–2.5 times higher than the dilution factor derived from single‐compound physical attenuation predictions, emphasizing that mixture interactions can enhance overall toxicity beyond additive predictions (Mallants et al., 2022). Thus, modeling frameworks can be extended to mixture contexts while considering combined effects rather than only individual species behavior.

Machine learning methods represent promising emerging approaches for predicting organic contaminant transport when mechanistic understanding remains incomplete or parameterization proves infeasible. Neural networks and related algorithms can identify patterns in complex datasets relating contaminant properties, soil characteristics, and environmental conditions to observed transport behavior (Gao et al., 2022). These approaches may prove particularly valuable for PPCP plant uptake, where empirical structure‐activity relationships can guide predictions across large numbers of compounds without requiring explicit mechanistic models. However, machine learning models need substantial training datasets and may perform poorly when extrapolated beyond the conditions represented in training data, limiting their application to novel contaminant mixtures or site conditions.

Machine learning applications extend beyond transport prediction to mixture toxicity assessment, where traditional concentration addition and independent action models face fundamental limitations. These classical approaches assume either similar or independent modes of action among mixture components, assumptions rarely satisfied by complex environmental mixtures containing thousands of chemicals with diverse toxicological targets (Cheng et al., 2024). Recent developments integrate non‐target screening analysis with machine learning algorithms to identify and semi‐quantify unknown chemicals in environmental samples, then predict their joint effects through trained models that can capture nonlinear interactions among mixture components. Such approaches have demonstrated utility for correlating bioavailability and toxicity of complex chemical mixtures in soils contaminated with petroleum hydrocarbons and metals, using empirical data including soil type, bioavailable concentrations, and exposure duration as input variables (Cheng et al., 2024). While these methods need substantial training datasets and careful validation, they offer pathways toward quantifying mixture effects when the identities and toxicities of all components remain unknown.

Integrated experimental‐modeling frameworks that combine laboratory characterization, field monitoring, and model development in iterative cycles offer pathways toward improved mixture predictions (e.g., Gaganis et al., 2004; see also Section 5). Rather than attempting to parameterize models entirely from laboratory data, these frameworks use field observations to constrain model parameters and test alternative conceptual models. Inverse modeling and data assimilation techniques allow systematic parameter estimation from field data while quantifying uncertainty. When applied to contaminant mixtures, these approaches can identify which mixture components and processes most influence overall behavior, guiding research priorities and monitoring designs.

## 5 OBSERVATIONS FROM COMTAMINATED SITES

Observations from contaminated sites provide essential validation of laboratory‐derived transport theories and reveal mixture behaviors under realistic complexity. The following contamination studies examine three sets of scenarios and illustrate how mixture complexity interacts with site‐specific factors to create transport patterns that diverge from single‐species predictions.

### 5.1 AFFF‐Impacted sites: PFAS mixtures

Decades of AFFF use at military installations, fire training areas, and industrial facilities have created widespread PFAS contamination impacting soils, vadose zones, and groundwater across the United States and internationally. These sites provide valuable opportunities to observe mixture behavior under field conditions with realistic complexity. AFFF formulations contain diverse PFAS compounds, often comprising >50 unique species ranging from 2 to 12 carbon atoms, including fluorotelomer‐based compounds, perfluoroalkyl sulfonamides, and numerous precursors alongside terminal perfluoroalkyl acids (McGarr et al., 2023). Following release, these mixtures infiltrate through vadose zones where sorption, transformation, and transport processes fractionate mixture compositions with depth and time (Figures 1 and 2).

One particularly instructive example comes from a military firefighter training area investigated by Nickerson et al. (2021). The site operated from approximately 1968 to 1991, during which personnel trained on proper firefighting techniques by igniting vehicles and equipment with hydrocarbon fuels within a shallow, unlined 36‐m diameter pit and then applying AFFF to extinguish the fires. Although AFFF applications ceased >26 years before sampling occurred in 2017, extensive high‐resolution characterization involving 105 soil samples and 58 groundwater samples across a 0.92 km^2^ area revealed persistent and substantial contamination. Total PFAS concentrations reached as high as 5.3 mg/L in groundwater at depths of 9–11 m below ground surface, demonstrating that vadose zone sources continue to supply underlying aquifers long after active contamination ends (Nickerson et al., 2021). Perhaps most striking was the finding that 47% of the total PFAS mass remained near the source zone despite decades of natural flushing, underscoring the challenge these sites pose for remediation. The study also documented pronounced differences in transport behavior among PFAS classes. Zwitterionic and cationic compounds composed up to 97% of the total PFAS mass in certain source area soils but exhibited limited lateral and vertical mobility compared to anionic species (Nickerson et al., 2021). This differential transport creates a scenario in which strongly sorbed precursor compounds act as lingering sources capable of transforming into more mobile perfluoroalkyl acids over extended timeframes. Evidence of such transformation was observed through changing PFAS composition along the groundwater flow path, including increasing concentrations of perfluorohexane sulfonamide downgradient from the source, suggesting degradation of parent sulfonamide‐based zwitterions (Nickerson et al., 2021).

Laboratory investigations using soils from AFFF‐impacted sites have helped elucidate the mechanisms controlling these persistent releases. Shea et al. (2025) collected vadose zone soils from an AFFF‐impacted site approximately 28 years after the final AFFF application and subjected them to controlled column experiments under varying saturation conditions. The experiments revealed that variably saturated conditions, which mimic natural wetting and drying cycles, dramatically enhanced PFAS leaching. For example, the average release rate of PFHxS increased by an order of magnitude under variably saturated conditions compared to constant saturation (Shea et al., 2025). Despite extensive flushing, however, substantial PFAS mass remained in the soils, with 51%–84% of PFOS retained due to rate‐limited desorption from solid phases. These findings carry implications for field‐scale predictions because vadose zones in natural environments rarely maintain constant water content but instead fluctuate with precipitation events and seasonal changes. The experiments corroborate the field‐scale relevance of transient saturation conditions discussed in Section 3.1 and suggest that climates experiencing frequent rain events may result in greater PFAS flux to underlying groundwater compared to regions receiving equivalent precipitation in fewer, larger events (Shea et al., 2025).

Complementary insights emerge from high‐resolution characterization of a deep vadose zone (∼106 m to groundwater) at an Arizona fire training facility, where 99% of PFAS mass remained concentrated within the upper 3 m despite approximately 50 years since initial AFFF application (Bigler et al., 2024). Spatial moment analysis revealed chromatographic separation following a sigmoidal pattern with molar volume, wherein intermediate‐chain PFAS exhibited differential migration depths while shorter‐chain compounds accumulated at the calcic horizon (100–140 cm below the soil surface). The authors attributed this accumulation pattern to limited deep infiltration characteristic of semi‐arid environments combined with potential enhanced retention from calcium carbonate‐rich soil properties. These observations highlight how regional pedogenic features common throughout the western United States may exert control on PFAS distribution patterns, representing an underexplored factor in vadose zone transport predictions.

McGarr et al. (2023) synthesized observations from several AFFF‐impacted fire training areas and identified complex spatial and temporal patterns in PFAS mixture composition that emerge from differential transport of various chain lengths and functional groups coupled with precursor transformation. One such site, the Ellsworth Air Force Base firefighter training area in South Dakota, operated from 1942 to 1990 and subsequently underwent groundwater remediation from 1996 to 2011 using oxygen injection wells (McGarr et al., 2023). Sampling conducted 21 years after the final AFFF release showed that the highest PFAS concentrations occurred in surface soils around the training area, consistent with the strong influence of air‐water interfacial adsorption observed at other sites. Interestingly, PFAS plume distribution in the sediments immediately above the water table followed the pattern of a co‐located volatile organic compound plume rather than displaying the chain‐length‐based partitioning expected from differential sorption. The authors attributed this unexpected pattern to enhanced precursor biotransformation during remediation, when oxygen injection stimulated microbial processes that accelerated the conversion of polyfluorinated precursors to terminal perfluoroalkyl acids (McGarr et al., 2023). This observation carries a cautionary implication for remediation design: oxygen injection during remediation efforts can accelerate biotransformation processes, but the enhanced formation of terminal degradation products may complicate overall risk reduction objectives if those products are themselves of regulatory concern.

### 5.2 Agricultural systems: PPCP and biosolids‐borne mixtures

Agricultural systems receiving biosolids applications or wastewater irrigation serve as long‐term natural laboratories for observing contaminant mixture transport under realistic field conditions. Spanning decades of repeated applications at many sites, these systems offer opportunities to observe accumulation patterns, breakthrough behavior, and natural attenuation processes that cannot be replicated in short‐term experiments. The documented evidence reveals a complex and sometimes contradictory picture, where site‐specific conditions exert profound influence on mixture fate.

Perhaps the most striking demonstration of deep PPCP penetration comes from Israel's Coastal Plain aquifer, where Zentner et al. (2015) investigated vadose zones beneath turfgrass irrigated with treated sewage effluents since the early 1960s. Drilling six boreholes through vadose zones ranging from 24 to 28 m thick across two fields, they detected nine of twenty target PPCP compounds at depths reaching 27 m. Carbamazepine concentrations reached 109 ng/kg, while caffeine accumulated at concentrations up to 36,700 ng/kg. Remarkably, venlafaxine, an antidepressant first permitted in Israel in 1998, had already penetrated the entire vadose zone at an average velocity of 2.8–4 m per year. The presence of clay layers containing up to 50% clay and soil organic carbon contents reaching 0.40% failed to prevent this deep penetration. These findings challenged the prevailing assumption that vadose zones effectively filter PPCP from infiltrating wastewaters, suggesting instead that irrigation with treated sewage effluents on aquifer recharge areas may pose risk to groundwater quality.

Yet the Israeli observations stand in marked contrast to those from wastewater‐irrigated agricultural soils elsewhere. Filipović et al. (2020) documented carbamazepine accumulation limited to the upper 60 cm after 20 years of irrigation with reclaimed wastewater. Their numerical simulations suggested that strong sorption to soil organic carbon initially limited downward transport, effectively trapping the compound in surface horizons. However, the modeling predicted a concerning future trajectory: once sorption capacity became saturated after 10–15 years of continuous loading, carbamazepine leaching toward groundwater would commence.

Lysimeter experiments in Germany offer additional insight into these contrasting behaviors. Thalmann et al. (2025) irrigated 1‐m sandy soil profiles with reclaimed wastewater containing ten organic micropollutants over three years. Their results revealed sharply divergent fates among compounds. The iodinated contrast agent diatrizoate exhibited near‐conservative transport, with 96% of applied mass leaching through the profile, while the short‐chain PFAS compound trifluoromethanesulfonate showed similarly high mobility at 66% leaching. These highly mobile compounds demonstrated tracer‐like transport owing to their inherently low sorption affinity under the prevailing soil pH conditions. In contrast, seven compounds, including carbamazepine, exhibited <2% leaching, suggesting effective retention or degradation within the biologically active topsoil. Notably, drainage occurred predominantly during winter months when evapotranspiration was minimal, while concentration spikes accumulated in soil pore water during vegetation periods when crop water uptake concentrated dissolved solutes.

Biosolids‐amended agricultural fields represent another important mixture scenario. Research sites with historical biosolids applications spanning decades often display accumulation of complex mixtures including PPCP, PFAS, microplastics, antibiotic resistance genes, and trace metals (Pozzebon & Seifert, 2023). Field monitoring at such sites reveals gradual accumulation of persistent contaminants in surface soils, while more mobile or degradable compounds show evidence of downward transport or attenuation. The presence of microplastics adds further complexity through potential co‐transport of sorbed organic contaminants, as discussed in Section 3.2.

### 5.3 Hydraulic fracturing sites: HFFA‐HFFC under extreme conditions

Settings for understanding HFFA‐HFFC mixture behavior in vadose zones are oil and gas production regions where documented hydraulic fracturing fluid spills have occurred. The Bakken Shale region of North Dakota exemplifies these extreme conditions. As one of the most intensively developed unconventional oil plays in North America, the Bakken region has experienced tens of thousands of documented spills over the past two decades, arising from well blowouts, pipeline failures, and storage facility releases (A. Klaustermeier et al., 2016; NDDEQ, 2021). Each incident released HFFA‐HFFC mixtures whose concentrations and compositions varied depending on reservoir characteristics, fracturing recipes, and production operations, creating a complex mosaic of contamination across the landscape.

Field investigations in the Bakken region have revealed that the extreme salinity of flowback and coproduced waters fundamentally reshapes vadose zone geochemistry in ways that influence virtually all subsequent HFFA‐HFFC transport processes. Electrical conductivity values often exceed 200 dS/m, with sodium chloride concentrations approaching saturation (Daigh & Klaustermeier, 2016; Klaustermeier et al., 2017; Green et al., 2020; A. Klaustermeier et al., 2016; Shrestha et al., 2017). Under such conditions, high sodium adsorption ratios trigger clay dispersion and soil structure degradation, which in turn reduces hydraulic conductivity and creates localized zones of impeded drainage (Green et al., 2020; Klopp & Daigh, 2020). These hydrological alterations not only affect the transport of HFFA‐HFFC constituents themselves but also compromise the effectiveness of subsequently applied remediation fluids. Perhaps more consequentially, such severe salinity inhibits microbial activity, limiting biodegradation of organic HFFA that might otherwise undergo natural attenuation (McLaughlin et al., 2016).

The impact of HFFA‐HFFC mixtures on soil microbial communities has been examined through controlled laboratory experiments that complement these field observations. Kookana et al. (2022) exposed five Australian soil types to hydraulic fracturing fluid and produced water, then monitored microbial responses over 60 days. Their findings revealed a striking contrast between the two fluids: HF fluid exposure completely inhibited nitrification in all tested soils, with no recovery observed throughout the experimental period, whereas three of five soils exposed to produced water showed complete nitrification recovery during the same timeframe. Intriguingly, although the biocides methylisothiozolinone and chloromethylisothiozolinone degraded rapidly with half‐lives of <2 days, microbial community structure remained significantly altered in HF fluid‐treated soils relative to controls at 28 days post‐treatment (Kookana et al., 2022). These findings carry implications for vadose zone transport prediction, as they indicate that HFFA‐HFFC mixture toxicity to nitrogen‐cycling microorganisms may impede the natural attenuation processes upon which transport models often rely.

The question of whether natural attenuation can adequately protect groundwater resources from HFFA‐HFFC contamination has received attention from both modeling and field perspectives, with findings that present a nuanced and sometimes contrasting picture. Mallants et al. (2022) developed a HYDRUS‐based simulation to examine natural attenuation potential for hypothetical flowback water spills in the deep vadose zones of the Beetaloo Sub‐basin in Australia's Northern Territory. Considering 63 organic and inorganic chemicals representative of flowback water, their model incorporated dilution, dispersion, sorption, biodegradation, and radioactive decay processes. The simulations suggested that deep unsaturated zones can indeed provide attenuation for most individual chemicals, reducing concentrations below ecological risk thresholds before reaching groundwater when vadose zone thickness exceeds 50–100 m. However, when Mallants et al. (2022) employed direct toxicity assessments to account for chemical mixture effects, the required safe dilution factor for 95% species protection proved 1.8–2.5 times higher than the dilution factor derived from physical attenuation processes alone, underscoring that mixture interactions can enhance overall toxicity beyond additive predictions.

Field‐scale observations from the Marcellus Shale region in Pennsylvania offer a complementary perspective on HFFA‐HFFC contamination potential. Shaheen et al. (2022) conducted a geochemical analysis of approximately 7000 groundwater samples and employed data mining techniques to explore correlations between groundwater chemistry and unconventional oil and gas development intensity. Their analysis identified localized hotspots in southwestern Pennsylvania where groundwater chloride and methane concentrations increased by 3.6 and 3.0 mg per liter, respectively, for each additional unconventional well drilled within the identified hotspot areas, with cumulative increases that could reach 43.2 mg/L for chloride and 21.0 mg/L for methane in the most densely developed hotspots, the latter exceeding the 10 mg/L explosion hazard threshold. While such correlations do not establish contamination at every well location, they suggest that localized incidents of brine migration or spillage may produce detectable effects on regional groundwater chemistry even where thick vadose zones might be expected to provide natural attenuation. Shaheen et al. (2022) further calculated that if brine contamination via wellbore leakage, impoundment failure, or surface spills represents the source, thallium concentrations could potentially exceed drinking water standards in the most densely developed hotspots, highlighting potential human health risks that extend beyond ecological endpoints. These field observations emphasize the importance of site‐specific assessments rather than reliance solely on regional attenuation predictions derived from modeling studies. Moreover, as discussed in Section 4.2, mixture toxicity assessments consistently show that combined effects of chemical cocktails require greater dilution than predicted from individual compound analyses, suggesting that real‐world complications including preferential flow paths and repeated spill events likely reduce natural attenuation capacity below model predictions for idealized homogeneous systems.

### 5.4 Overarching implications from contaminated site studies

The field and laboratory investigations described in Sections 5.1 through 5.3 reveal several overarching patterns that transcend specific contaminant classes and site conditions. Most fundamentally, vadose zones consistently function as persistent secondary sources that sustain groundwater contamination through slow desorption, precursor transformation, and remobilization during transient flow events. Groundwater monitoring at AFFF‐impacted sites consistently shows persistent PFAS plumes decades after initial releases, sustained by these vadose zone processes (McGarr et al., 2023; Nickerson et al., 2021; Shea et al., 2025). Similarly, PPCP accumulation in agricultural vadose zones receiving biosolids or wastewater irrigation creates long‐term reservoirs that gradually release contaminants to underlying aquifers. These observations carry an important practical implication in that removing dissolved groundwater contamination without addressing vadose zone sources will likely prove ineffective for long‐term plume control.

Competition among mixture components for finite sorption sites emerges as a consistent mechanism influencing transport across all three contaminant classes. For PFAS, competitive sorption at air‐water interfaces and solid surfaces enhances mobility of certain species while retarding others, creating chromatographic separation effects that alter mixture composition with depth. Similar competitive dynamics likely operate for PPCP and HFFA‐HFFC mixtures, though they remain less thoroughly characterized. Spatial heterogeneity in soil properties and preferential flow paths creates site‐specific transport patterns that challenge extrapolation across locations, necessitating integrated characterization of contaminant distributions, soil properties, and hydrological conditions at scales relevant to transport processes.

Predicting mixture behavior requires careful consideration of multiple interacting factors, including soil properties, application or release history, compound‐specific physicochemical characteristics, and climate‐driven hydrological dynamics. Mixture interactions including competitive sorption, co‐facilitated transport, and combined toxicity effects consistently produce behaviors that diverge from single‐species predictions, validating the need for mixture‐focused research approaches in vadose zone science.

## 6 KNOWLEDGE GAPS AND RESEARCH PRIORITIES

In the preceding sections, we have proposed research priorities based on what we identified as numerous limitations in our current understanding of contaminant mixtures in vadose zones. These research priorities are organized sequentially below into six thematic areas based on our perspective of their importance for advancing mixture science and their potential to yield actionable knowledge for environmental management.

### 6.1 Mixture sorption and competitive transport

Systematic characterization of mixture sorption and transport across chemical classes represents the most fundamental research need for advancing predictive capabilities. While substantial literature exists describing sorption behavior for selected PFAS, PPCP, and petroleum hydrocarbons individually, comparatively little research has examined competitive sorption in realistic mixtures containing representatives from multiple contaminant classes. Critical questions remain regarding whether PFAS and PPCP compete for the same sorption domains or partition independently to distinct sites, how variable charge conditions in soils affect competitive sorption of differently charged species, and whether generalized frameworks relating contaminant properties to mixture sorption behavior can enable predictions for unstudied compounds. Answering these questions will need systematic experimental programs examining sorption across ranges of mixture compositions, soil properties, and solution chemistries, combined with development of mechanistic sorption models parameterized from molecular properties rather than empirical fitting alone, but still validated with focused laboratory experiments. Priority should be given to mixtures representative of common contamination scenarios, including AFFF formulations, wastewater‐derived PPCP cocktails, and biosolids‐borne contaminant assemblages.

Additionally, soil co‐contamination with inorganic and organic pollutants along with particulate matter is increasingly recognized as the norm rather than the exception, yet current research predominantly examines single pollutants or groups of similar compounds (Y. Chen, Cao et al., 2025). Understanding how coexisting pollutants alter each other's physicochemical behavior and bioavailability through competitive sorption, facilitated transport, and modified transformation pathways represents a priority for mixture‐focused vadose zone research. Recent field investigations at PFAS manufacturing sites have revealed that PFAS contamination can alter soil DOM composition at the molecular level, with PFAS promoting transformation of DOM components toward protein‐like compounds and the aromaticity of DOM influencing the vertical migration of perfluoroalkyl sulfonates through soil profiles (Y. Chen, Cao et al., 2025). These bidirectional interactions between PFAS and DOM introduce additional complexity to mixture transport predictions and suggest that DOM characterization may be important for understanding PFAS fate in contaminated soils.

### 6.2 Transformation products and reaction pathways

Transformation products deserve greater research attention than they have received to date. Many emerging contaminants undergo partial transformation in environmental systems, generating products that may exhibit comparable or greater toxicity and mobility than parent compounds. Current analytical methods typically target parent compounds with limited attention to transformation products, creating blind spots in our understanding of mixture evolution. Strong research efforts should identify relevant transformation products for priority contaminants, develop analytical methods for their quantification, characterize their physicochemical properties and environmental behavior, and assess their toxicity relative to parent compounds. This work necessitates integration across analytical chemistry, environmental chemistry, microbiology, and toxicology to achieve comprehensive understanding. Advancing characterization of HFFA‐HFFC environmental fate would benefit from adoption of risk‐based screening frameworks that prioritize chemicals exhibiting persistence, mobility, and toxicity properties, as these substances pose particular concern for drinking water resources (Jin et al., 2022). Such approaches would direct analytical and research resources toward compounds most likely to reach receptors while remaining hazardous, rather than attempting exhaustive characterization of all mixture components. High‐resolution mass spectrometry coupled with suspect and non‐target screening methods offers pathways toward more comprehensive identification of transformation products, including halogenated byproducts formed under the elevated halide concentrations characteristic of flowback waters. Because persistent and mobile substances can pass natural attenuation barriers and accumulate within drinking water resources, their identification and elimination from HF formulations represents an important component of proactive water quality protection strategies. Additionally, particular emphasis should be placed on PFAS precursor transformation pathways, pharmaceutical metabolites in vadose zones, and products formed through abiotic reactions under extreme geochemical conditions such as those characteristic of hydraulic fracturing spills.

### 6.3 Field‐scale validation studies

Field‐scale transport studies in natural vadose zones represent a complementary priority needed to validate laboratory findings and test model predictions under realistic complexity. Most mixture transport research relies on laboratory column experiments using repacked soils under controlled conditions. While valuable for elucidating fundamental processes, these experiments cannot capture the full complexity of structured soils, preferential flow, spatial heterogeneity, transient boundary conditions, and coupled biogeochemical processes operating at field scales. Instrumented field sites with detailed monitoring of mixture composition at multiple depths and times, combined with characterization of hydrological and geochemical conditions, would provide datasets suitable for testing mixture transport theories and constraining model parameters.

Appropriate monitoring technologies, including tension lysimeters, suction cups, wick lysimeters, and passive capillary samplers, enable collection of soil solution samples for temporal and spatial characterization of mixture composition (Singh et al., 2017). Quantifying PFAS mass discharge from vadose zones to groundwater requires integration of porewater concentration measurements with site‐specific groundwater recharge estimates, and recent work has developed tiered approaches for estimating recharge rates at PFAS‐impacted sites (Newell et al., 2023). Such frameworks enable assessments of the relative contributions of vadose zone sources to groundwater contamination, informing decisions about remediation prioritization and the potential applicability of monitored natural attenuation strategies. Selection among these approaches depends on site‐specific conditions, target analyte properties, and sampling frequency, with recent developments in passive sampling methods offering advantages for minimizing disturbance while capturing representative pore water compositions.

Suction lysimeters have emerged as important tools for direct measurement of PFAS porewater concentrations in vadose zones; however, their application requires careful attention to potential PFAS interactions with lysimeter materials, sampling effects related to air‐water interfaces encountered during sample collection, and the limited sampling area relative to site heterogeneity (Costanza et al., 2025). Laboratory validation and consideration of soil‐texture‐based sampling issues with lysimeters are essential for ensuring representative samples, particularly given the air‐water sorption tendencies of PFAS compounds. Such sites could be established at existing contaminated locations or created through controlled release experiments, though the latter approach faces logistical and regulatory challenges for emerging contaminants. Field studies should explicitly examine transient flow effects, preferential pathway contributions, and natural soil structure influences on mixture transport (i.e., nonequilibrium flows), as these factors consistently emerge as critical yet understudied controls on contaminant behavior.

### 6.4 Multi‐mechanism remediation technologies

Remediation strategies capable of addressing multiple contaminant classes need substantial development and testing. Most existing remediation technologies target specific contaminant types using mechanisms optimized for those chemicals. For example, activated carbon injection effectively treats certain organic contaminants but may prove ineffective for highly polar species (Lenka et al., 2024). When complex mixtures span diverse properties, no single technology may adequately address all components. Developing sequential or hybrid approaches that leverage multiple mechanisms simultaneously represents important applied research.

A fundamental conceptual question underlying remediation research is whether contaminants should be transported downward through leaching or extracted upward through surficial removal. Conventional approaches for saline HFFA‐HFFC contamination rely on amendments and irrigation to leach salts below the root zone, but this strategy merely redistributes contaminants deeper into vadose zones where they may eventually reach groundwater or resurface through capillary rise (Green et al., 2020). Novel surficial approaches, including crystallization inhibitors that induce harvestable salt efflorescence and evaporative wicking materials that accumulate salts at the soil surface, offer permanent contaminant removal from vadose zone systems rather than redistribution (Daigh & Klaustermeier, 2016; A. W. Klaustermeier et al., 2017; Green et al., 2022). Whether analogous upward extraction principles could apply to PFAS or PPCP mixtures, perhaps leveraging their affinity for air‐water interfaces under controlled evaporative conditions, represents an unexplored research direction.

Combining surficial extraction technologies with electrokinetic methods may offer synergistic remediation potential. Electrokinetic remediation applies low‐intensity direct current to induce electromigration of charged species and electroosmotic flow of pore water toward electrodes and has demonstrated effectiveness for metals and certain polar organic contaminants in fine‐textured soils where hydraulic methods prove ineffective. Coupling electrokinetics with surface extraction could direct contaminant migration upward toward collection zones rather than toward subsurface electrodes, potentially enabling recovery of mobilized species. Furthermore, integrating in situ chemical oxidation or biostimulation with electrokinetic transport could transform recalcitrant parent compounds into more polar, ionizable degradation products that exhibit enhanced electromigration. Such integrated approaches warrant investigation for PFAS mixtures, where precursor transformation generates anionic perfluoroalkyl acids with increased mobility under applied electric fields, and for PPCP mixtures containing ionizable pharmaceuticals. The diversity of PFAS species present at contaminated sites, spanning different chain lengths and functional groups with varying sorption behavior, likely necessitates staged remediation sequences targeting different PFAS subclasses rather than single mechanism approaches.

Integration of remediation research across PFAS, PPCP, and HFFA‐HFFC could identify transferable principles, enabling more efficient management of sites contaminated with multiple emerging contaminant classes. Priority should be given to field demonstrations at biosolids‐amended agricultural soils, AFFF‐impacted vadose zones, and hydraulic fracturing spill sites where mixture complexity presents particularly severe remediation challenges.

### 6.5 Integrated mixture toxicity assessment

Integrating mixture toxicity into transport predictions represents a step toward realistic risk assessment. Risk assessments typically evaluate individual contaminants against compound‐specific standards or guidelines, implicitly assuming independence among mixture components. However, toxicological studies consistently demonstrate mixture effects, including concentration addition, synergy, and antagonism that alter overall toxicity relative to individual compound predictions (Escher et al., 2020). The primary challenge for mixture risk assessment lies not only in accounting for synergy but also in identifying and quantifying the numerous unknown or uncharacterized chemicals contributing to overall mixture toxicity. Developing frameworks to couple vadose zone transport models with mixture toxicity assessments would enable more realistic risk evaluation than current approaches permit. This integration faces challenges including limited toxicity data for emerging contaminants, uncertainty regarding relevant exposure scenarios, and computational demands of coupled modeling. Beyond toxicity modeling, accurate risk assessment requires understanding which mixture components are actually bioavailable to receptors. Current risk frameworks typically rely on total contaminant concentrations without explicitly accounting for partitioning and bioavailability dynamics that govern actual exposure (Cipullo et al., 2018). These oversights may contribute to overestimation of ecotoxicological effects while limiting the efficacy of risk frameworks to inform targeted remediation strategies. Bioavailability should be understood not as a fixed concentration but as a dynamic process between organisms and chemical uptake over time, influenced by soil properties, contaminant aging, and mixture composition (Cipullo et al., 2018). Integrating bioavailability assessments into mixture risk evaluation offers pathways toward more realistic and cost‐effective site management, though standardized methods applicable to complex chemical mixtures remain underdeveloped. However, recent advances in both transport modeling and mixture toxicology make this goal increasingly achievable. Initial efforts should focus on high‐priority receptor pathways, including groundwater used for drinking water supplies and plant uptake in agricultural systems receiving wastewater or biosolids, where mixture exposures are well‐documented and health concerns are established.

Achieving integrated mixture assessment will require evolution of regulatory frameworks beyond compound‐specific approaches. Proceedings from recent scientific meetings note the growing opinion that successful environmental risk assessment for contaminated soils should evaluate direct and indirect effects at the systems level, formulate holistic protection goals for soil organisms, incorporate mixture toxicity through additive risk calculations, and establish feedback loops between prospective modeling and retrospective field monitoring (Kotschik et al., 2025). Current regulatory approaches that parcel complex environments into scenarios addressing single compounds, model organisms, and highly specific uses most often disregard the reality of chemical mixtures in soils and fail to capture cumulative effects on ecological communities. Terrestrial model ecosystems containing natural communities of soil organisms and trait‐based approaches linking species sensitivity to contaminant modes of action offer promising methodologies for scaling risk assessment from individual species to community and ecosystem levels (Kotschik et al., 2025). Adapting these approaches from surface soil applications to vadose zone environments presents additional challenges related to oxygen gradients, moisture variability, and depth‐dependent biological communities, yet represents an important direction for developing protective management frameworks.

### 6.6 Climate change and evolving land use impacts

Understanding how climate change and evolving land uses will affect mixture dynamics in vadose zones represents a longer term research challenge with growing urgency. Changing precipitation patterns alter infiltration rates and saturation conditions, affecting both transport and transformation processes. Increasing frequency of droughts and floods creates more extreme transient conditions with potentially larger impacts on air‐water interface processes and colloid mobilization. Extreme weather events, including intense precipitation, flooding, and drought cycles, affect contaminant fate through multiple mechanisms, including altered volatilization rates, mobilization of previously bound contaminants through changing redox conditions, accelerated leaching during intense rainfall, and concentration effects during drought (H. Chen, Gao et al., 2025). Integration of climate variables into mixture transport models represents an emerging priority as extreme weather frequency increases. Expansion of wastewater reuse for irrigation due to water scarcity concerns will increase applications of contaminant mixtures to agricultural lands. Coastal areas face saltwater intrusion that may alter sorption behavior and microbial activity in vadose zones. Adaptation of vadose zone management strategies to these changing conditions will need predictive frameworks that can anticipate mixture behavior under future scenarios. While immediate research emphasis should remain on understanding mixture behavior under current conditions, building flexible modeling frameworks and establishing long‐term monitoring programs will position the scientific community to address climate‐related challenges as they emerge.

## 7 CONCLUSIONS

This review synthesizes current understanding of complex contaminant mixtures in vadose zones, emphasizing PFAS, PPCP, and HFFA‐HFFC as representative emerging contaminant classes. Field observations consistently demonstrate that mixture effects, including competitive sorption, co‐facilitated transport, and altered transformation dynamics, produce behaviors that diverge substantially from single‐species predictions. Air‐water interface processes exert control on PFAS transport, particularly under the transient saturation conditions characteristic of natural vadose zones. Vadose zones function as persistent secondary sources sustaining groundwater contamination through slow release processes and precursor transformation. Current modeling frameworks, while increasingly sophisticated, face substantial challenges in representing the complex physicochemical interactions and field‐scale heterogeneity characteristic of mixture‐contaminated vadose zones. Successful application of existing models requires extensive parameterization that often proves infeasible for the dozens to hundreds of compounds present in environmental mixtures.

Advancing vadose zone science toward more realistic and protective frameworks also requires sustained investment in mixture‐focused research. Priorities include systematic characterization of competitive sorption across chemical classes, field‐scale validation studies under realistic complexity, comprehensive investigation of transformation products and their environmental fate, development of multi‐mechanism remediation technologies, and integration of mixture toxicity into transport predictions. Rather than attempting exhaustive characterization of all compounds present in mixtures, the research community should develop generalizable frameworks relating contaminant properties to environmental behavior, identify indicator compounds representing broader classes, and focus resources on mixture components driving overall risk. To make meaningful progress, interdisciplinary collaboration is needed to bring together expertise in analytical chemistry, environmental engineering, microbiology, toxicology, and modeling.

From regulatory perspectives, mixture effects challenge existing frameworks that address individual contaminants through compound‐specific standards, necessitating evolution toward approaches that account for combined exposures and toxicity. The environmental and human health stakes associated with contaminant mixtures in vadose zones justify the investment needed to advance mixture‐focused science and bridge the gap between single‐species research and the complex reality of environmental contamination.

## AUTHOR CONTRIBUTIONS

**Aaron Lee M. Daigh**: Conceptualization; data curation; investigation; visualization; writing—original draft; writing—review and editing.

## CONFLICT OF INTEREST STATEMENT

The author declares no conflicts of interest.

## Data Availability

No data were generated in this review.
